# Frequency of the Statistical Methods and Relation with Acceptance Period in Archives of Iranian Medicine Articles: A Review from 2015–2019

**DOI:** 10.34172/aim.2022.43

**Published:** 2022-04-01

**Authors:** Farzan Madadizadeh, Sajjad Bahariniya

**Affiliations:** ^1^Center for healthcare Data modeling, Departments of biostatistics and Epidemiology, School of public health, Shahid Sadoughi University of Medical Sciences, Yazd, Iran; ^2^Departments of Health Care Management, School of Public Health, Shahid Sadoughi University of Medical Sciences, Yazd, Iran

**Keywords:** Archives of Iranian Medicine, Statistical data interpretation, Statistics

## Abstract

**Background:**

Statistical methods (SM) are a ubiquitous tool in research. This study aimed to review SM used in original article published in the *Archives of Iranian Medicine* (AIM) and assess their effect on article acceptance period.

**Methods:**

The original articles published in the period 2015–2019 from volumes 18 to 22 and issues 1 to 12 of the AIM were reviewed and six items such as SM, study design, statistical population, sample size, software and acceptance period were extracted. Mean (SD), frequency (percentage) and multiple response analysis (MRA) were used for description. The Kruskal-Wallis test and Spearman correlation coefficient were used for data analysis in SPSS 26 with significance level at 5%.

**Results:**

During the study period, 423 original articles were reviewed. The statistical population in most of them was patients (38.8% and 164 articles), and most studies (51.5% and 218 articles) had a sample size of less than 500 people. The study design in most of the articles was analytical-observational (55.1% and 233 articles), and 79.7% (337 articles) used SPSS for data analysis. The median (IQR) acceptance period was 194 (134.25). MRA results showed that the highest rate of use of SM was related to descriptive statistics (277 articles, 30.3%) and Chi square test (130 articles, 14.2%). In the last two years, the acceptance period had a declining trend. There was no significant relation between mentioned variables and acceptance period (*P*>0.05).

**Conclusion:**

Contrary to the researchers’ misconceptions, the acceptance period was not affected by SM, study design, statistical population, sample size, or type of software.

## Introduction

 Statistical methods are recognized as essential and vital tools in biomedical research. In modern medical research, the use of advanced and extensive statistical methods is very important. Improper use of statistical methods can reduce the quality of biomedical research, and lead to poor interpretation and wrong conclusions. On the other hand, the use of complex and inefficient statistical methods solely for the purpose of superficial improvement of the study can be considered as an immoral approach.^1-5^

 Medical journals with a wide coverage of various topics in the field of medicine and public health have many enthusiasts around the world. In Iran, the *Archives of Iranian Medicine* (AIM) is a valuable journal and a reputable clinical resource that has been working since 1998 and has been publishing articles monthly since 2012. The journal welcomes biomedical experiences and clinical research on common diseases in the region, as well as analysis of factors that may moderate the management of diseases and related medical problems. The impact factor (IF) of this journal in 2019 was about 0.996 and the 5 years IF was 1.46, It also had an H-index of about 45 and a CiteScore of about 2.20.

 Printing high quality articles is the main goal of all journals, especially journals in the field of medical sciences. To achieve this goal, it is necessary to evaluate the articles published in the journal, review the quality of articles, identify strengths and weaknesses of the journal and eliminate problems and shortcomings in future issues.^6^

 The use of advanced statistical methods works as a double-edged sword that, on the one hand, leads to deeper analysis and more valuable findings, and on the other hand, makes it difficult to interpret data; so the presence of biostatistics experts can be helpful at this stage. Improper use of statistical methods can lead to a loss of researchers’ effort and a waste of time and investment. One of the key factors to improve the quality of medical journals is the acceptance of articles that have used valid, advanced and effective statistical methods. Reputable international journals that seek promotion, in order to increase the quality of the articles and to ensure the validity of the statistical methods used in the articles, conduct detailed reviews by judges with statistical expertise. The type of statistical methods used in articles may play an important role in attracting the attention and decision of the journal editors regarding the acceptance or rejection of articles.^7-10^

 Some authors believe that if they use more advanced statistical methods instead of just conventional and simple methods, they will have a better chance of publishing the article and will spend less time in the judging process. So, according to this belief, they start with using advanced statistical methods and sometimes get stuck in interpreting the outputs of these methods, and finally submit their article with incorrect interpretations to the journal. On the other hand, knowledge of the variety of statistical methods used and the trend of the average refereeing time, type of study, statistical community and applications can be useful for self-evaluation and promotion of the journal and identify gaps in science and increase the variety of articles. In this regard, review articles have recently been published focusing on the statistical methods used in the original articles of scientific journals.^11-16^

 Due to the importance of the content mentioned in the previous paragraphs, the present study aimed to identify and review statistical methods, sample size, study design, statistical population, and type of software used in the original articles and investigate their relationship with the acceptance period (interval between the received and accepted dates mentioned in the articles) of articles published in the Archives of Iranian Medicine in the period 2015 to 2019. Statistical review of journal articles is usually done over a period of 5 or 10 years.^3,4^ At the time of conducting this study (June 2020), AIM’s 2020 issues were not yet complete, so this year was not included in our study. Therefore, the period from 2015 to 2019 was selected as our study period.

## Materials and Methods

 This review study was conducted in June 2020 on AIM Journal articles. All original articles published in a period of 5 years (2015–2019) from volumes 18 to 22 and issues 1 to 12 of the Archives of Iranian Medicine were reviewed. Each article was reviewed by a three-member team consisting of a biostatistics expert and two expert researchers in the field of medical research. Six variables such as statistical methods, sample size, study population, software, study design and acceptance period were extracted from the articles.

 In order to perform descriptive statistics, considering that it was possible to use more than one method in each article, the multiple response analysis (MRA) technique was used and the frequency and frequency percentage were reported. MRA is one of the valuable statistical methods for analyzing questions with the possibility of more than one answer. In the output of this method, which we used in the present study, unlike simple descriptive statistics tables, a table is provided containing two absolute/relative frequencies, one for the sum of responses and one for cases. In our study, the answers were the tests used in articles and the cases were original articles. In order to investigate the relationship between the type of statistical methods, software, statistical population and type of study with the acceptance period of the articles, first the normality of errors in quantitative variables was investigated by the Kolmogorov-Smirnov test and after rejecting the null hypothesis and confirmation of non-normality in errors, the Kruskal Wallis test and Spearman correlation coefficient were used. Statistical analyses were performed using SPSS version 26 with a significance level of 5%.

## Results

 A total of 742 articles were published during the study period, of which 423 (57%) were original articles that were included in our study. Other published articles such as Case Reports, Reviews, Letters to the Editor, Editorials, Brief Reports, Opinions, Book Reviews, Systematic Reviews and others were not included in the study (Table 1).

**Table 1:** Types of Articles Published During 5 Years

**Type of Articles**		**2015**	**2016**	**2017**	**2018**	**2019**	**Total**
Original Article		81	94	114	64	70	423
Case Report		37	20	5	7	14	83
Brief Report		3	3	2	1	3	12
Photoclinic		4	5	5	7	6	27
Letter to Editor		8	11	7	11	12	49
Editorial		2	-	1	-	-	3
Review		9	11	4	4	5	33
History of Medicine in Iran		13	5	9	4	4	35
Systematic Review		4	6	4	2	9	25
Opinion		-	1	3	5	1	10
Mini Review		1	-	-	1	-	2
Round the World		1	-	-	-	-	1
Report		-	-	4	1	1	6
Book Review		1	-	-	2	-	3
Event		1	2	1	-	-	4
Obituary		2	4	1	-	-	7
Research Methods		1	2	1	2	1	7
Guideline		-	-	1	-	-	1
Protocol Design		-	-	1	-	-	1
Cohort Profile		-	-	-	-	1	1
Meeting report		-	-	-	-	1	1
Author’s Reply		-	-	-	-	5	5
Study Protocol		1	1	-	1	-	3
**Total**		169	165	163	112	133	742
**Acceptance period**	**Median (days)**	**	**	**	194	190.5	194
	**IQR (days)**	**	**	**	130.5	152.5	134.25

**Not available: From 2015 to 2017, the time of receiving and accepting the article was not mentioned on the first page.

 All statistical methods used in the original articles published in the Archives of Iranian Medicine during the study period of (5 years: 2015–2019) are summarized in Table 2.

**Table 2:** Statistical Methods Extracted from the Articles of 2015–2019

**Methods**		**Brief Description**		**Acceptance Period**	
		**Simple Methods**	**Complex Methods**	**Median (days)**	**IQR (days)**
Parametric test	Descriptive statistics	mean, standard deviation, percentage and frequency, minimum and maximum, median, Interquartile range, DALY, QALY		180	101
	T test	Independent, paired *t* test	**-**	175	128.5
	Regression	Linear regression	Logistic regression	210	79
	ANOVA-ANCOVA	One-way ANOVA	Two-way ANOVA, repeated measures ANOVA	258	118
	Multivariate Analysis	**-**	All type of factor analysis, multivariate regression, MANOVA, ICC, SEM/GSEM	162	105.25
	Pairwise comparison test	**-**	SNK, LSD, Duncan multiple range test, Bonferroni correction, Tukey	247	243
	Normality tests	**-**	Shapiro-Wilks, Kolmogorov-Smirnov	251	116.75
	Assumption checking test	Leven test	**-**	160.5	112.25
	Nominal variable test	McNemar test	Cochran Q test	94	64
	Chi square test	Fisher exact, Hardy Weinberg, Wald test	Trend analysis	231	189
	Correlation analysis	Pearson correlation, Spearman, Kendall (Mann-Kendall)	Partial	74	40
	Survival analysis	Kaplan-Meier, Log rank test	Cox regression^*^, Parametric models (Weibull, Exponential, distribution), Risk analysis	210	108
Non parametric test		Kruskal-Wallis test, Mann-Whitney U test, Wilcoxon Test	Friedman Test	195	147

DALY, Disability-adjusted life year; QALY, Quality-adjusted life year; MANOVA, Multivariate analysis of variance; ICC, Intraclass correlation coefficient; SEM, structural equation modeling (description GSEM fits generalized SEMs); SNK: student-Neuman-Keuls; LSD, Least significant difference. *Semi-parametric.

 In the period of the present study (from 2015–2019), the statistical population reported in the articles mostly consisted of patients (164 cases, 38.8%) and human samples at the community level (155 cases, 36.6%). The least use pertained to statistical data with 1.4% (6 cases); the results of the Kruskal-Wallis test showed that the mean acceptance period in different statistical populations was not significantly different (*P* = 0.148) (Table 3).

**Table 3:** Statistical Population of the Reviewed Articles

**Statistical Population**	**N**	**%**	**Acceptance Period**		***P *** **Value**
			**Median (days)**	**IQR (days)**	
Patients	164	38.8	181	124	0.148
Community-level samples	155	36.6	200	161.75	
Laboratory samples	44	10.4	164	151.75	
Occupations	22	5.2	210	108.50	
Statistical data	6	1.4	162	158	
Others	32	7.6	171	154.50	
Total	423	100.0	194	134.25	

**Community-level samples** included women, men, mothers, girls, adults, people over 15, children, students, infants, smokers, the elderly and middle-aged, families, adolescents, young people, urban and rural residents, Blood donors, healthy people in the community, obese people, prisoners, couples, spouses and other similar cases. **Laboratory samples** included laboratory animals such as mice and rabbits, genes, cells, chromosomes, serum samples, blood samples, water samples, cord samples, microbial samples, bacteria, viruses, strains. Vaccines, human tissue samples, suspected leishmaniasis lesions, RNA, DNA and the like. **Occupations** included physicians, health care providers, hospital staff such as nurses, specialists, experts, staff, workers, drivers, and the like. **Statistical data (secondary)** included information, documents and reports. **Other statistical populations** included websites, drugs, suicide attempts, ovarian and brain tumors, teeth, mortality, research units, articles, etc.

 During the study period, most studies had a sample size of less than 500 people (51.5%, 218 cases). In 12.3% (52 cases) of the studies, the sample size was unknown; the results of the Kruskal-Wallis test showed that the mean acceptance period in different sample sizes was not significantly different (*P* = 0.356) (Table 4).

**Table 4:** Sample Size Used in the Reviewed Articles

**Sample Size**	**N**	**%**	**Acceptance Period**		***P*** ** Value**
			**Median (days)**	**IQR (days)**	
Less than 100	129	30.5	215	152	0.356
100-500	89	21.0	162	110.50	
500-1000	27	6.4	226.5	160.25	
More than 1000	126	29.8	210	138.50	
Unknown	52	12.3	152	96	
Total	423	100.0	194	134.25		

 The study design in most articles was analytical-observational (233 cases, 55.1%) and only 3.3% (14 cases) were qualitative; the results of the Kruskal-Wallis test showed that the mean acceptance period in different study designs was not significantly different (*P* = 0.439) (Table 5).

**Table 5:** Study Design in the Reviewed Articles

**Study Design**	**N**	**%**	**Acceptance Period**		***P*** ** Value**
			**Median (days)**	**IQR (days)**	
Descriptive	105	24.8	173	139.75	0.439
Analytical-observational	233	55.1	205	132.25	
Analytical-interventional	71	16.8	117.5	156.25	
Qualitative	14	3.3	166.5	143	
Total	423	100.0	194	134.25	

**Descriptive:** descriptive cross-sectional, pilot study, case series study, comprehensive study, quantitative study, exploratory descriptive study. **Analytical-observational:** cross-sectional study, descriptive analytic cross-sectional, analytic cross-sectional, case-control study, cohort study, retrospective study, prospective study, observational study, longitudinal study, ecological study. **Analytical-interventional:** clinical trial study, experimental study, semi-experimental study, interventional study, histologic study, histopathological study.

 Based on the results, most articles had selected the SPSS software for analysis (337 cases and 79.7%); the results of the Kruskal-Wallis test showed that the mean acceptance period in different types of software was not significantly different (*P* = 0.072) (Table 6).

**Table 6:** Statistical Software Used in the Reviewed Articles

**Name of Software**	**N**	**%**	**Acceptance Period**		***P*** ** Value**
			**Median (days)**	**IQR (days)**	
SPSS software	337	79.7	199	134.25	0.072
STATA software	35	8.3	205	210.25	
MAXQDA software	10	2.4	140	84.50	
GraphPad Prism software	12	2.8	178	147	
R Software	7	1.7	170	167	
AMOS software	4	0.9	223	149	
Others	12	2.8	138	168	
Unknown	6	1.4	162	77.50	
Total	423	100.0	194	134.25	

**Other software included**: PASW software, SAS software, MedCalc statistical software, Mutation Surveyor software, Sequencing Analysis software, GenEX software, Genetic Analyzer Software, Arc Map ver. 10.3, BUGS 3.2.3 Software, Statistical Software, Epi Info^TM^ 7 statistical package, Gene Marker V1.97, LinRegPCR (12.x) software, CodonCode Aligner 5.0.1, MEGA software, Excel and others.

 The results of our review showed that the time of receiving and accepting the article and mentioning it in the main file of the article was done exactly since the first issue published in 2018. The results showed that the mean (SD) and median (IQR) acceptance period of the articles were 200.99 (106.856) and a 194 (134.25) days, respectively.

 The results of the Kruskal-Wallis test showed that there was a significant difference in the acceptance period across months (*P* = 0.001), such that the longest time (541 days) pertained to the second month of 2018, after which the acceptance period diminished and reached 180 days at the end of 2018. Also, the declining trend continued and reached 164 days at the end of 2019. There was no significant relationship between the acceptance period and the study design (*P* = 0.568), statistical population type, (*P* = 0.148), type of software (*P* = 0.072), or type of statistical methods (*P* = 0.252). The results of the Spearman correlation coefficient test did not show a significant relationship between sample size and acceptance period (r = 0.13, *P* = 0.482).

**Figure 1:**
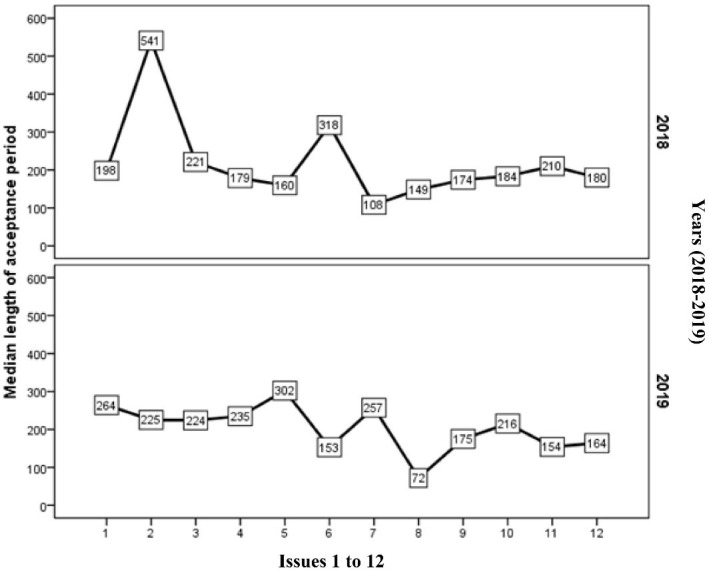
Median Length of Acceptance Period During 2 Years Based on Issues 1 to 12.

Figure 1 shows the trend of the acceptance period of articles reviewed in our study. There was a downward trend during both years.

 The findings of MRA showed that generally, among all the statistical methods that were used in the articles, the highest rate of use pertained to descriptive statistics (30.3% and 227 cases), chi-square test (14.2% and 130 cases), *t* test (12.6% and 115 cases), and regression models (11.5% and 105 cases). Among all articles, 68.9% used descriptive statistics, 32.3% used chi-square test, 28.6% used *t*test, 26.1% used regression models, 19.2% used one-way analysis of variance, and 18.9% used non-parametric methods (Table 7).

**Table 7:** Results of Multiple Response Analysis

**ID**	**Statistical Methods**	**All Methods**		**Percent of All Articles**
		**N**	**%**	
1	Descriptive statistics	277	30.3	68.9
2	T test	115	12.6	28.6
3	Chi-square test	130	14.2	32.3
4	Regression	105	11.5	26.1
5	ANOVA-ANCOVA	77	8.4	19.2
6	Non parametric tests	76	8.3	18.9
7	Post hoc tests	25	2.7	6.2
8	Normality tests	40	4.4	10.0
9	Correlation analysis	31	3.4	7.7
10	Survival analysis	21	2.3	5.2
11	Multivariate analysis	8	0.9	2.0
12	Nominal variable tests	5	0.6	1.2
13	Assumption checking test	3	0.4	0.7
	**Total**	**913**	**100.0**	**227.1**

**Figure 2:**
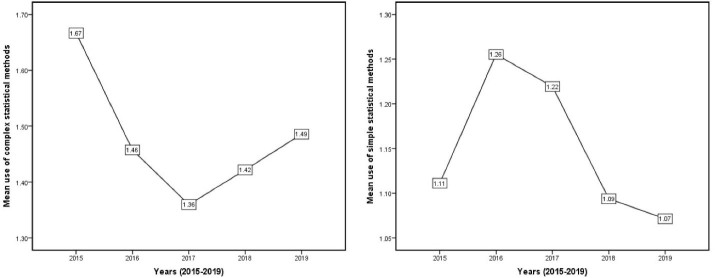
Mean Number of Times to Use Complex and Simple Statistical Methods During 5 Years.

 The trend of using simple statistical methods (such as descriptive statistics, *t* test, chi-square, Fisher’s exact test, McNemar, one-way analysis of variance, Mann–Whitney U test, Kruskal-Wallis test) and advanced statistical methods (ANCOVA, MANOVA, COX regression, multiple regression, SEM etc.) is presented in Figure 2. The average use of advanced statistical methods increased during the years under review.

## Discussion

 In recent years, the philosophy of using statistical methods in biomedical research has undergone massive changes. Meanwhile, some researchers believe that if they use more sophisticated and advanced statistical methods to analyze research data, they will have a better chance to publish the article in a short time.

 The findings of this study, which were obtained by reviewing the AIM journal in the period 2015 to 2019 and based on 423 original articles, showed that patient and community-level samples (including women, men, mothers, girls, adults, children, students, infants, smokers, the elderly and middle-aged, families, adolescents, young people, urban and rural residents, transplant recipients, blood donors, healthy people in the community, obese people, prisoners, couples, spouses and others) were the most frequent statistical population used in the reviewed articles. This shows the special attention of the leading AIM journal to accepting articles on the topics of diseases and problems in the community. Due to the possibility of missing statistical data, despite the great effort of researchers, the manuscript may be rejected by reviewers. So, according to the results of the present study, the lowest frequency of statistical populations pertained to statistical data such as information, documents and reports.

 The cumulative frequency in Table 4 shows that 36.2% of the sample sizes were more than 500 people. It may be thought that the larger the sample size of the study, the less time it will take for the article to be accepted. The results of our study showed that this assumption is wrong and there was no relationship between the acceptance period and the article’s sample size.

 The findings of the present study in mentioning the type of study design in the studied articles showed that most of the study designs were analytical-observational. Up to 55% of the studies were analytical-observational. This confirms the special attention of AIM to this type of studies. Qualitative studies involve the collection and analysis of non-numerical data to understand concepts, opinions, or experiences. These studies are used to gather in-depth insights into an issue or generate new ideas for research. According to the results, only 3.3% of the studies were qualitative. Given that qualitative research can play a major role in health research, it requires more attention from the editors of the journal to this issue.^17^

 SPSS is one of the most popular statistical analysis software among researchers. Although it has very good coverage of common statistical methods, it is not a complete software, and researchers must use programming software or semi-programming software to use more advanced statistical methods. The findings of the present study showed that among the articles published in this journal, the majority of articles used SPSS and a smaller percentage of published articles used programming (such as R, Win Bugs, Matlab, Python, etc.) or semi-programming software (such as Minitab, Stata, etc.).^18^ A possible reason for this could be the features of being user-friendly and the availability and easy learning of SPSS.

 According to Table 7, the MRA results showed that among both the total tests and the articles, the highest use of statistical methods pertained to descriptive statistics and the chi-square test. In the present study, the Fisher exact test, Hardy-Weinberg, Wald test and Trend analysis were considered as the chi-square test. Frequent use of statistical tests in the findings of similar studies^19-21^ in line with our study showed that the most common statistical method used in the articles pertained to descriptive statistics and the chi-square test.

 The chi-square test is one of the most important statistical tests used to evaluate the relationship between two qualitative variables.^20^ This test is one of the available statistical methods, user-friendly and easily used in most statistical software. Therefore, it has many fans. Our results showed that the use of advanced and specialized statistical methods in 2019 was reduced compared to previous years. This requires the attention of editors to accept articles with more advanced statistical methods.

 Based on the results of the present study, from the last issue published in 2017, the time of receipt and the time of acceptance are mentioned on the first page of the articles. Mentioning the time of receiving and accepting articles in the published articles is very important and in order to promote AIM journal, it is necessary for the editors of the journal to pay attention to such issues.

 Accuracy in judging and accepting articles in a short period of time will pave the way for a journal to move forward. On the other hand, the authors tend to submit their articles to journals that have a shorter acceptance period. In general, as shown in Figure 1, fortunately, the time of acceptance of articles by AIM had a declining trend during 24 months, and this issue, along with the accuracy of referees, can pave the way for the future progress of the journal.

 The results of the Kruskal-Wallis test showed that there was no significant difference in the median time of acceptance of articles in terms of the type of test, sample size, type of statistical community, study design and type of software. This highlights the misconception of those researchers who believe that by using more advanced statistical tests, a larger sample size, more sensitive statistical populations, more sophisticated study designs, and more advanced statistical software can publish their article in a shorter time. The results of the present study were consistent with the results of similar studies.^22^

 In terms of strengths, this study examined and analyzed more variables compared to similar studies. One of the main limitations of this study was lack of access to articles rejected by the journal and only accepted articles were reviewed. Otherwise, based on statistical analysis of logistic regression, we could calculate the odds of the acceptance of an article based on each of the variables studied in this study, such as sample size, type of statistical method, type of statistical population, etc. Another limitation was lack of access to information on the refereeing time of all articles (accepted and rejected), which, if accessible, we could have definitely performed a more valuable analysis. To calculate the time required to publish the articles, the difference between the time of receipt and the time of acceptance that was listed on the first page of the article, was considered.

 In conclusion, it took about 28 weeks from submission to acceptance in AIM journal. Given that the acceptance period is an important item for researchers to choose a journal and on the other hand, it can be a key item to promote journal indexing, it seems to be beneficial to reduce it and more attention is required from the editors of the journal. Most of the articles were of the observational-analytical design with patient as the statistical population. The statistical methods of most articles consisted of simple tests. The frequency of advanced statistical methods was decreasing during the study period. Acceptance of articles with advanced statistical methods seems necessary. Contrary to what the researchers thought, the type of test, sample size, type of community, type of study design or type of software were not effective factors regarding the acceptance period.
